# 

**Published:** 1891-05-23

**Authors:** 


### NOTICE TO CORRESPONDENTS.

All MS., Letters, Books for Review, and other matters intended for the Editor should be addressed The Editor, The Lodge, Porchester Square, London, W.

The Editor cannot undertake to return rejected M.S., even when accompanied by stamped directed envelope.

**Notice to Advertisers and Subscribers.**

All Advertisements, Orders for Copies of The Hospital, and Business Communications should be addressed to The Publisher, *not to the Editor*, The Hospital, 140, Strand, W.C.

The Cost of Subscription, post free, is as follows:—Per week, 1½d.; per quarter, 1s. 8d.; per half-year, 3s. 3d.; per annum, 6s. 6d. Foreign:—Per annum, 8s. 8d.; payable, in all cases, in advance.

**NOTICE.—The Index for Vol. IX.. (ending March, 1891) is on sale at the Office of this Journal, price 2½d., post free.**

### General Charities.

[This Column is devoted to an account of work of charitable institutions other than Medical Charities. The Editor will be glad to receive reports or news of work at 140, Strand. *Philanthropy* should be plainly written on the envelope.]

The income received by the **RAGGED SCHOOL UNION** during the past year, though it shows a slight increase over the previous year, is none too large for the many-sided work and claims of the Institution. During last year 4,004 children, elder scholars, and adults, were sent for a fortnight to the eight holiday homes of the Union, consisting of seaside and country cottages, and 10,000 children have revelled in the luxury of a day in the country. In addition to this a large number of meals were given, and new and used clothing distributed during the winter months. Subscriptions and donations will be gratefully received by the Secretary, Mr. John Kirk, 13, Exeter Hall, Strand, W.C.

A great event in the year at the **FARNINGHAM AND SWANLEY ORPHAN HOMES** is the May Festival, which has taken place, and which is an opportunity for visitors to see the children both at work and play. The chief feature of the festival, of course, is the crowning of the May Queen, who is chosen from one of the Sunday Schools which contribute to the support of the Homes which form a small and compact village. We regret to learn that an increased income of £2,000 a-year is urgently needed to prevent the Committee of this old-established Institution from having to curtail any portion of its useful work. Donations will be gratefully acknowledged by the Secretary, Mr. Benjamin Clarke, Bank Buildings, Ludgate Circus, E.C.

The annual meeting of the **CHILDREN'S FRESH-AIR MISSION** took place on May 13th. Last year this society sent away 2,632 little children for a holiday, gathered chiefly from the districts of Holborn, Clerkenwell, and St. Luke's. Gifts of clothing will be gratefully received to enable the children to go away tidily dressed. It seems a pity that this fund, although working with the Children's Country Holiday Fund, does not amalgamate with it. Differences in organization ought to be easily overcome, and extra children could be sent away with the money which is now spent on the printing, stamps, &c., of a separate society. Subscriptions will be gratefully received by the Hon. Treasurer, Mr. Walter Hazell, St. Peter's Schools, Onslow Street, Clerkenwell Road, E.C.

The Society for establishing **PLAYING FIELDS FOR LONDON**, which has recently held its first annual meeting, has yet to win its spurs, though its objects are such as are likely to find it many supporters. The primary object is to provide "assisted" education in cricket and give pent-up Londoners an opportunity of enjoying healthy recreation near home. Few can afford the time, even if they can give the money, to go far afield for their out-door exercise, and, having the knowledge of this in view, the Society hopes to supply new playing-fields near the populous centres of the town. According to Sir Henry James, it was calculated 15 years ago that every year the addition to the population of the metropolis represented the whole of the inhabitants of a greater city than Oxford. Thus the yearly addition to London of some 80,000 or 90,000 inhabitants must mean the consequent destruction of open spaces. In addition to the physical difficulties with which the committee have to contend they are not free from another embarrassment in the form of want of funds to promote their objects. They have an opportunity of doing much lasting good by giving their poorer fellow citizens a chance of some wholesome relief from the monotonous routine of a city life if the public will help them with money.

May finds the societies for sending London children into the country for a holiday hard at work collecting funds, issuing reports, and generally rousing up the public to an interest in their labour of love, for such it undoubtedly is. **THE CHILDREN'S COUNTRY HOLIDAY FUND** have had a successful annual meeting, presided over by Princess Beatrice, and their record of last year's work is a splendid one, for nearly 24,000 little Londoners had a fortnight's country holiday, thanks to their efforts. The society is spreading over London rapidly, and this year three new districts are being organized—Fulham, Stratford, and Stoke Newington. London is ever growing, and so the necessity for extension of the work increases every day. And it is not only pecuniary help that is wanted, but personal labour—labour which requires much tact and patience, and in the poorer neighbourhoods it is difficult to find people with leisure enough to arrange the details for despatching the children. The welfare of little children has been a common meeting-ground for every shade and divergence of religious opinion. Sectarianism is unknown at the Children's Country Holiday Fund, and the list of Vice-Presidents is a guarantee of this. The names of the Bishop of London, Dr. Allon, Dr. Adler, the Chief Rabbi, and Cardinal Manning show how easy it is for men to sink prejudice and unite in a common good. "Unless a considerable sum is in hand by the beginning of June, the work of the summer is seriously hindered." This is a little note attached to the report, the truth of which is obvious, and we who have watched the difference in the children from the time when they arrive in the country depressed and weary, to the day when they go home again, happy and filled with fresh thoughts and ideas of life, strongly appeal for funds to be sent to the Secretary, 10, Buckingham Street, Strand, W.C.

### Scraps and Gleanings.

The ports of Rio Janiero and Pernambuco continue to be infected with yellow fever.

The Skinners' Company have sent a donation of £20 to the Irish Distressed Ladies' Fund.

The Irish Distressed Ladies' Fund has received a donation of £25 from the Clothworkers' Company.

The Princess Louise will pay a visit to the Metropolitan Hospital, Kingsland Road, on Thursday, the 28th inst.

Dr. C. W. Marriot has resigned his position of Honorary Surgeon to the Warneford Hospital on account of his health.

The Friendly Societies of Chichester held a church parade on Sunday, the 10th inst., in aid of the funds of the Infirmary.

The Canongate Hospital is being prepared by the Edinburgh authorities in case influenza should become epidemic in that town.

The London Skin Hospital, 47, Cranbourne Street, W.C., will, next month, be removed to larger premises, at 40, Fitzroy Square.

Several cases of leprosy have occurred in British Columbia. The disease was probably brought into the country by the Chinese.

A Bal Poudre, in aid of the National Society for the Prevention of Cruelty to Children, will be held in Queen's Gate Hall, on June 1st.

A boy named David Wilkinson, living near Spalding, has committed suicide while suffering from influenza. He was only fifteen years of age.

The Clothworkers' Company have given a donation of £250 to the Extension Fund of the Maria Grey Training College, which now amounts to £7,742.

The Derby Infirmary Saturday Fund Committee have decided to appeal to the working classes of the town and county to aid the fund for rebuilding the Derbyshire Infirmary.

In consequence of the prevalence of the influenza among members of Parliament, it is proposed that the House should be thoroughly fumigated during the Whitsuntide holidays.

The Rev. Prebendary Whittington presided at the annual festival of the City of London Truss Society, at the Albion Tavern. During the evening donations amounting to £760 were announced.

The various members of the Birmingham and District Licensed Victuallers' Beer and Wine Trade Associations have contributed £905 18s. 9d. towards the fund for building the new infirmary.

The Convalescent Home of the Chelsea Hospital for Women, at West Hill, St. Leonards-on-Sea, will be opened by the Countess Cadogan on June 3rd. A special train will leave Charing Cross at 11.15 a.m.

Mr. Samuel Pryer, of Chipping Norton, has received an offer of £1,000 towards erecting a cottage hospital for the borough, the only condition being that the institution shall be commenced within twelve months.

The influenza epidemic is still very severe in Birmingham, though it is fortunately not productive of a very high rate of mortality. No fewer than 600 new cases have been reported at one hospital in a single day.

Subscriptions amounting to over £2,000 were announced at the festival dinner in aid of the funds of the North-Eastern Hospital for Children, which was held under the presidency of the Duke of Connaught on the 15th inst.

At the last sitting of the Royal Commission on Vaccination, Mr. Maskell, vaccination officer of the Leicester Union, was examined respecting the statistics of small-pox patients in his district. Mr. Hopwood, Q.C., Recorder of Liverpool, also gave evidence.

The Lord Mayor presided at the seventh annual meeting of the National Society for the Prevention of Cruelty to Children. The report stated that the Society had warned 5,054 of cruelty, and had prosecuted 963, 92 per cent. being convicted. The income has risen from £8,871 to £19,421.

The 18th annual report of the Wigan Royal Albert Infirmary, states that 1,164 in-patients, and 5.604 out-patients were treated during 1890. The receipts amounted to £17,329 18s. 4d., of which £10,071 11s. 6d. has been added to the endowment fund. The expenditure of the year was £8 434 8s. 8d.

Sir Richard Quain presided, on the 14th inst., at the distribution of prizes, medals, and certificates to the pupils of University College in the faculty of medicine. Sir Richard gave an interesting address to the students, and exhorted them above all things to regard their profession as a generous and noble art, and not as a trade.

On Thursday last Mr. Harding-Cox and Miss Olga Garland gave a special matinée of "Caste," at Terry's Theatre, in aid of the Hospital for Sick Children, Great Ormond Street. Robertson's comedy was followed by a new and original musical comedietta, entitled "Sweepstakes," written and composed by Mr. Ernest Lake.

A crowded meeting was held at the Hotel Metropole on the 12th inst. on behalf of the Children's Country Fund. Twenty-three thousand, seven hundred and seventy-one London children have been sent to enjoy village life by the society, the parents contributing £5,245, or 35 per cent. of the total expenditure. Princess Beatrice presided at the meeting.

The sixtieth annual report of the Coventry Provident Dispensary states that the number of members admitted during 1890 was 1,298. The amount received from members, which in 1889 was £2,949 4s. 10d., was last year £3,073 11s. 11d. A legacy of £100 has been received, and has been placed to the reserve fund, the balance of which was last year increased to £446 19s. 11d.

The report for 1890 of the Nottingham and Midland Eye Infirmary states that 3,354 new cases were treated during the year, which is the largest number since the establishment of the institution. The total income was £776 16s. 9d., which included three legacies. The expenditure was £537 19s 1d. Increased interest in the welfare of the hospital has been shown by the working classes.

### BOOKS RECEIVED.

THIN, JAMES, Edinburgh.

"The Intra-Cranial Circulation and its Relation to the Physiology of the Brain." James Cappie, M.D. Price 7s. 6d.

WRIGHT AND CO., Bristol.

"The Medical Annual and Practitioner's Index for 1891."

### THE STUDENT OF MEDICINE.

[All communications for this Supplement should be addressed to The Editor of The Hospital, at 140, Strand, with the words, "Students' Column," written in the left hand top corner of the envelope.]

### APPOINTMENTS.

At King's College, London, T. G. Brodie, M.R.C.S., L.R.C.P., has been appointed joint demonstrator of physiology. At the Guest Hospital, Dudley, J. H. Wilkinson, M.R.C.S., L.R.C.P., has been appointed resident medical officer. At the Queen's Hospital, Birmingham, George St. Johnston, M.R.C.S., L.R.C.P., has been appointed house physician. At the West Riding Lunatic Asylum, Wakefield, Ernest A. Shaw, B.A., M.B., C.M. Camb., has been appointed pathologist and anæsthetist. At the Hospital for Sick Children, Newcastle-on-Tyne, Walter W. Heelas, M.R.C.S., L.R.C.P., has been appointed resident medical officer. At the National Institution for the Blind, Leeson Park, Dublin, Richard Thomas Hearn, M.D., has been appointed physician. At the Oxford Eye Hospital, R. Edie, M.B., C.M. Edin., has been appointed house surgeon. At the Royal Victoria Patriotic Asylum for Girls, Wandsworth Common, Arthur E. Dobson, M.R.C.S., L.R.C.P., L.S.A., has been appointed medical officer. At the Hospital for Consumption, Brompton, Sidney Allden, M.D. Durh., has been appointed resident medical officer. At the Western Skin Hospital, Great Portland Street, P. S. Abraham, M.D., M.A., B.Sc., F.R.C.S.I., has been appointed honorary physician, and F. J. Allan, M.D., clinical assistant.

### VACANCIES.

The following vacancies are announced this week, the amount of salary in each case being given after the name of the institution:

### Resident Medical Officers.

Blackburn and East Lancashire Infirmary, Junior House Surgeon—Salary £50, with board, &c.

City of London Hospital for Diseases of the Chest, Victoria Park, E., House Physician—Board, &c.

General Hospital for Sick Children, Manchester, Junior Resident Medical Officer—Salary £80, with board, &c.

Royal Westminster Ophthalmic Hospital, House Surgeon.

### Non-Resident Medical Officers.

Cancer Hospital (Free), Fulham Road, Surgeon; also Honorary Pathologist.

Eccles and District Medical Association, Assistant Medical Officer.

General Hospital, Birmingham, Honorary Surgeon.

Royal Berks Hospital, Reading, Physician.

University of Glasgow, Assistant Examiner in Physiology—Annual fee £30.

### STUDENT'S BOOKSHELF.

### Elementary Human Physiology.*

This little work forms one of the series known as "Hughes's Science Text-Books." In the preface the author states "This book meets the requirements of teachers and others who may be reading for the Science and Art Department subject of Animal Physiology. It will also be useful to medical students during their first year, as a text-book for the ante-primary examination of the Conjoint Board." As an elementary work on the subject of Animal Physiology it is certainly wonderfully clear and well written, the arrangement of the various sections is excellent, and great pains have been taken with the illustrations, which have been executed by Mr. W. H. Harvey. The book commences with a short introduction to the great subject of Animal Physiology, and the next section deals with the Chemistry of the Body. The third is on the Anatomy of the Body, in which are given original diagrams of the various bones forming the skeleton and diagrams showing the circulatory and digestive systems, &c. Chapter IV. is entitled "The Histology of the Body, or Minute Structure of the Tissues." Commencing with the element of the body, a single cell, the author then goes through all the more important forms of epithelial cells, connective tissues, cartilages, bone, muscle, and nerve fibres, on to the more complex ganglia and lymph cells, the important points about each one being clearly defined, and accompanied by remarkably clear diagrams. Chapter V. is on the Functions of the Body as a whole, VI. is on the Blood, and VII. on the Organs of Circulation, in which some admirable drawings, illustrating the cavities of the

* "Elementary Human Physiology." By Walker Overend, B.A. Oxon., B.Sc. Lond., late Scholar of Balliol College and Radcliffe Travelling Fellow, Deputy Lecturer on Physiology at St. George's Hospital, London, W. (Joseph Hughes and Co., Pilgrim Street, Ludgate Hill, London, E.C.)

### The Hospital Advertiser.

THE STUDENT OF MEDICINE—(*continued*).

heart and the course of the flow of blood through them, are given. The circulation in the arteries is first discussed, also with the help of diagrams, then that in the capillaries, and, finally, the circulation through the veins. Chapter VIII., on Respiration, is accompanied by some very clear diagrams of a very difficult subject for the student to grasp, namely, the mechanism of the movements of the ribs by the muscles which act upon them. The drawings which are given in this section are, however, so clear and explicit that there will be no trouble for the beginner to fully understand the mechanism of respiration. Chapter IX. is on Foods: their Digestion and Absorption, and in this, with regard to vegetarianism, Mr. Overend writes: "The advocates of vegetarianism assert truly that plants contain all the three essential food-stuffs; that is, proteids, as the gluten of flour, fats in the form of oils and starch. But vegetable proteids are not so nutritive as those of flesh, since not more than one-half is utilised in the intestines, and the remainder leads to disturbances of digestion and disinclination for food. It has been boastingly maintained that beans, which contain 23 per cent. of a vegetable proteid termed legumin, are as valuable as meat. But the percentage of this proteid assimilated is very low, and when combined with bacon, as is usual in a mixed diet, beans form an article which can only be tolerated by individuals with the most vigorous digestive powers." Chapter X. deals with Excretion, the Skin, and Kidneys, and XI. with the Joints, Mechanism of the Skeleton, the Heat of the Body, and the Larynx. Here again a very difficult section of physiology, the mechanism of the skeleton, is very clearly put with the guidance of some clever diagrams, such as the three orders of levers as illustrated by muscles in the body. The section devoted to the Larynx is also fully illustrated. The last chapters are on the Central Nervous System and the Special senses. The diagrams and descriptions of the course of the fibres in the cord are good, so also the derivation of the cranial Nerves from the different cerebral hemispheres in the course of development of the brain are clearly shown. The diagram of the attachments, and of the actions of the different ocular muscles in the chapter on the eye is especially descriptive. The last chapter is devoted to some practical exercises, &c., and a table of the weights and measures of some of the more favourite examination questions is also given. There is a very good and full index, and the whole book is very well printed in large type, and all the important words in each section are printed in capitals and not in italics, as is so often the case with text-books; so also all the sections are numbered. Of this small book as a whole we have nothing but praise to bestow, and the author has produced a book which will be a great boon to students commencing physiology, and although the substance matter is purely elementary in itself, yet the whole subject is put forward with that scientific accuracy and detail, which is usually so conspicuously absent in elementary works of this kind. Again, another point in favour of the book is that the author has not endeavoured to popularise the subject of physiology by writing in a popular strain, and so appealing to badly educated minds, but has maintained a high standard of technical precision throughout.

### SCHOLARSHIPS AND PRIZES.

### Bristol General Hospital.

Lady Haberfield entrance scholarship (value thirty guineas), G. B. Price; Clark surgical scholarship (value fifteen pounds, for proficiency in surgery, open to students of the third year), A. E. Milner; Sanders scholarship (value twenty-two pounds ten shillings, for proficiency in medicine, surgery, and obstetric medicine, open to students of the fourth year), T. M. Carter; Martyn Memorial pathological scholarships (each of the value of ten pounds, for proficiency in pathology and morbid anatomy)—April, J. B. Bate; October, J. Freeman and T. M. Carter (equal).

### Bristol Royal Infirmary.

First entrance scholarship (value thirty-five guineas), A. E. H. Pinch; second entrance scholarship (value ten guineas), E. A. Dorrell; Suple surgical prize (a gold medal and seven guineas in money, open to students of the third year), W. R. Hadwin; Clark prize (value fifteen guineas, awarded to the student in his third year of study at the Infirmary and Medical School who most distinguishes himself in the class examinations), W. R. Hadwen; Tibbits Memorial prize (value nine guineas, for proficiency in practical surgery), H. J. Mole; obstetric prize (value three guineas, in books), T. Pitt; pathological prizes (each of the value of three guineas), T. Pitt, C. Wintle, H. S. Jackson, A. St. John, and C. Bernard.

### NOTES FROM THE SCHOOLS.

### Edinburgh.

The Summer Session began on May 4th at the University and School of Medicine.

The Clinical examinations for the M.B. final began on May 1st, and will be continued on into June.

### THE STUDENT OF MEDICINE—(continued).

The following gentlemen have been appointed residents:—*Medical:* Professor Grainger Stewart; A. C. E. Gray, M.B., C.M.; Professor Fraser; R. M. Ronaldson, M.B.C.M., M.R.C.S.; Professor Greenfield; P. C. Evans, M.B., L.R.C.P. and S.; Professor Simpson; C. C. Douglas, M.B., C.M.; Dr. Muirhead; H. G. Melville, M.B., C.M.; Dr. Wyllie; A. S. Duncan, M.B., C.M.; Dr. Affleck; F. L. Brown, M.A., M.B., C.M.; Dr. Brackenridge; R. P. Mackenzie, M.B., C.M.; Dr. Halliday Croom; G. Aitchison-Robertson, M.D. *Surgical:* Professor Annandale, W. C. Smith, M.B., C.M., and J. W. Dowden, M.B., C.M.; Professor Chiene; R. M. Horne, M.B., C.M.; Mr. Duncan, J. A. Menzies, M.B., C.M.; Mr. Miller; W. Carmichael, M.B., C.M.; Dr. Maclaren; C. B. Ker, M.B., C.M.

In the second professional examination a very large percentage of candidates seem to have been referred, many being refused an oral.

The University has conferred the honorary degree of LL.D. on Dr. John Beddoe, B.A., F.R.S., on account of his eminence as a physician, as a scientist, and especially as an anthropologist; and on Sir George Murray Humphry, F.R.S., of Cambridge.

VANS DUNLOP SCHOLARSHIPS.—The Vans Dunlop Scholarship in Chemistry and Chemical Pharmacy, of the annual value of £100, and tenable for three years, has been awarded to William Cossar MacKenzie, D.Sc. The Vans Dunlop Scholarship in Anatomy, Physiology, Materia Medica, and Pathology, has been awarded to Robert Hutchison.

Professor Sir William Turner gave the first of a series of anthropological demonstrations on the skull on May 15th.

### London School of Medicine for Women.

Two more students of the London School of Medicine and the Royal Free Hospital have recently received appointments. Miss Annette M. Benson, M.B., B.Sc.Lond., has been appointed resident clinical assistant to the Paddington Infirmary, and Miss J. B. Henderson, L.R.C.P., and S.Edin., has been promoted from the post of resident clincial assistant to the Holloway Sanatorium to that of third assistant medical officer. Miss Benson was a distinguished Girton student. She took honours in medicine at the M.B. examination of the University of London last November. The Worshipful Company of Clothworkers has made a grant of £50 to the alterations fund of the London School of Medicine for Women.

### PASS LISTS.

### University of Cambridge.

The following degrees have been conferred:—

DOCTORS OF MEDICINE.—James Kerr, St. John's; Benjamin Bloomfield Connolly, Gonville and Caius; Herbert Timbrell Bulstrode, Emmanuel; and Philip Hicks, Cavendish Hostel.

BACHELORS OF MEDICINE AND BACHELORS OF SURGERY.—Geoffrey Edward Hale, King's; Charles Percival White, Clare; Ernest Alan Robert Newman, Gonville and Caius; and Edward Surman Peck, Christ's.

BACHELOR OF SURGERY.—Benjamin Bloomfield Connolly, Gonville and Caius.

### University of Durham—Faculty of Medicine.

At the Convocation held on April 28th the following were recommended for the degree of Doctor in Medicine for Practitioners of fifteen years' standing: Norman Bruce Elliot, F.R.C.S. Eng.; John Hamilton, F.R.C.S., L.R.C.P. Edin., L.F.P.S. Glasg.; Fredk. K. Marsh, L.R.C.P. Edin., L.F.P.S. Glasg.; Willoughby Furner, F.R.C.S., Eng.

The following were recommended for the degree of M.D.: George Berwick, M.B., B.S. Durh.; Henry Luther Ewens, M.B., B.S. Durh., M.R.C.S., L.R.C.P. Lond.; Alfred John Gregory, M.B., B.S. Durh., M.R.C.S., L.S.A.; Francis Hy. Mead, M.B., B.S. Durh., M.R.C.S.; George Newstead, M.B., B.S. Durh.; Charles Patrick O'Connor, M.B. Durh., M.R.C.S.; Nathan Raw. M.B., B.S., L.S.Sc. Durh.; Louis Robinson, M.B. Durh., M.R.C.S., L.S.A.; Jas. Scott Tew, M.B., B.S. Durh., M.R.C.S., L.S.A., D.P.H. Camb.

The following were recommended for the degree of Bachelor in Medicine (M.B.):—

HONOURS (SECOND CLASS).—Edwid Cecil Willcox, College of Medicine, Newcastle-on-Tyne.

PASS-LIST.—Walter Reyner Brunton and Wm. Arthur Rudd, London Hospital; John Clay, Alfred Cox, Arthur Jas. Dale, Thomas Hartley, Wm. Cyril Haswell, John Dobson Wardale, Algernon Edward Luke Wear, Robt. Anthony Welsh, College of Medicine, Newcastle-on-Tyne; Henry Wm. James Cook, Charing Cross Hospital; Ramsay Lamy Daly, Chas. Forsyth, and Arthur John Gardner, M.R.C.S., L.R.C.P., Yorkshire College, Leeds; Robert Dunmore Hotchkis, M.A., St. Bartholomew's Hospital; Joseph Wood, Owens College, Manchester.

The following were recommended for the degree of Bachelor in Surgery (B.S.): Percy Rutherford Adkins, M.B.Durh., St. Thomas's Hospital; Frederic Carden Brodie, M.B. Durh., M.R.C.S., L.R.C.P., L.S.A., Middlesex Hospital; Henry Wm. James Cook, Charing Cross Hospital; John Clay, Alfred Cox, Arthur James Dale, William Cyril Haswell, George Grahamsley Howitt, M.B. Durh., John Dobson Wardale, Algernon Edward Luke Wear, Robt. Anthony Welsh, and Edward Cecil Willcox, College of Medicine, Newcastle-on-Tyne; Ramsay Lamy Daly and Chas. Forsyth, Yorkshire College, Leeds; Harry Fowler, M.B. Durh., Owens College, Manchester; Robt. Dunmore Hotchkis, St. Bartholomew's Hospital; and Joseph Woods, Owens College, Manchester.

### THE STUDENT OF MEDICINE—(*continued*).

### Royal College of Physicians of London.

The following gentlemen, having passed the necessary examinations, were, at the ordinary comitia of the College on April 30, admitted Licentiates: Messrs. George Stewart Abram, University of Cambridge and University College Hospital; Alexander William Allan, Charing Cross Hospital; John Ernest Cecil Allott, Guy's Hospital; William Rushton Ashworth, Westminster Hospital; Percy James Atkey, St. Thomas's Hospital; George Walter Barber, Middlesex Hospital; Frank Whinfield Bartlett, University College Hospital; Bertram George Mortimer Baskett, General Hospital, Bristol; James Curling Bates, Guy's Hospital; Basil Boake, Dublin; Charles Richard Box, St. Thomas's Hospital; William George Brett, Guy's Hospital; Reginald Brown, St. Bartholomew's Hospital; James Busfield, Charing Cross Hospital; Albert Carling, Charing Cross Hospital; Richard Clegg, Owens College and Royal Infirmary, Manchester; Edward Beresford Collings, Yorkshire College and General Infirmary, Leeds; Richard Hawtrey Collins, Charing Cross Hospital; Arthur Reginald Colyer, Charing Cross Hospital; Thomas Edward Constant, Charing Cross Hospital; Albert Corner, St. Bartholomew's Hospital; Edward Cornish, Guy's Hospital; Ernest Richard Haines Cory, London Hospital; Cuthbert Bracey Dale, St. Bartholomew's Hospital; Anthony Dalzell, St. Thomas's Hospital; David Livingstone Davies, Owens College and Royal Infirmary, Manchester; Cecil Lacy Dawson, St. Bartholomew's Hospital; Henry Castriot de Renzie, Westminster Hospital; Appu Hennedige de Silva, Durham University; Robert Beauchamp Dudgeon, University College and Royal Infirmary, Liverpool; John Constable Ellis, St. George's Hospital, George Beaumont Featherstone, Guy's Hospital; Ernest James Finch, St. Mary's Hospital; Frank Chubb Ford, St. Bartholomew's Hospital; Arthur Claude Fox, London Hospital; John Francis, Middlesex Hospital; Louis Arthur Francis, St. Mary's Hospital; John Freeman, General Hospital, Bristol; William Frederick Gardener, St. Thomas's Hospital; John Guest Gornall, St. Thomas's Hospital; Denis Gratwicke Halsted, London Hospital; Edward Thornborough Harden, University College Hospital; Edward Drummond Hay Hawke, Charing Cross Hospital; Arthur Hawley, Queen's College and Hospital, Birmingham; Alfred George Hebblethwaite, Yorkshire College and General Infirmary, Leeds; Charles Edward Milnes Hey, St. Mary's Hospital; Augustus Westinghausen Hoffman, Charing Cross Hospital; George Waddington Holton, Owens College and Royal Infirmary, Manchester; John Armistead Home, St. Bartholomew's Hospital; Arthur Talman Ilott, Charing Cross Hospital; Henry Daniel James, King's College Hospital; Leo Edward James, Westminster Hospital; Alfred Jeffreys, St. Thomas's Hospital; Frederick Johnson, St. Bartholomew's Hospital; Henry Thomas Jones, St. Thomas's Hospital; John Furneaux Jordan, Queen's College and Hospital, Birmingham; Charles Stanley Kirton, London Hospital; Herbert Knevitt, London Hospital; Charles Ewbank Lansdown, St. Mary's Hospital; Arnold Lawson, Middlesex Hospital; George Dalton Nugent Leake, St. George's Hospital and Royal Infirmary, Manchester; Francis Edward Marshall, King's College Hospital; Edward Charles Masser, Queen's College and Hospital, Birmingham; William Frederick Edward Milton, St. Thomas's Hospital; William James Olivey, St. Thomas's Hospital; Charles Sumner Patterson, Edinburgh University and Middlesex Hospital; Charles Marten Powell, St. Bartholomew's Hospital; Rhys Powell, King's College Hospital; Arthur Edward Price, St. Thomas's Hospital; Alfred Theodore Rake, Guy's Hospital; James Rannie, Aberdeen University; Charles Kinsman Rawes, Owens College and Royal Infirmary, Manchester; Horace Richardson, Guy's Hospital; Alonzo George Rider, University College Hospital; Montague Louis Bouchier Rodd, Middlesex Hospital; William Gusterson Rogers, Guy's Hospital; Frank Mortimer Rowland, Cambridge University and Queen's Hospital, Birmingham; Arthur Rudd, Westminster Hospital; Leonard George Scudamore, St. Thomas's Hospital; Charles Sempill de Segundo, St. Bartholomew's Hospital; Bruce Gordon Seton, St. Bartholomew's Hospital; Ernnest Newlyn Smith, University College Hospital; James Richardson Spensley, London Hospital; William Dewing Spurrell, Guy's Hospital; George Robert Fabris Stilwell, St. Thomas's Hospital; Walter John Erneley Sumpter, University College Hospital; Francis Edward Swinton, St. Bartholomew's Hospital; Ernest Frank Syrett, St. Bartholomew's Hospital; William Henry Alisoun Tebbs, Westminster Hospital; Abraham Thomas, Guy's Hospital; Reginald Thorpe, St. George's Hospital; Hugh Stanley Thurston, St. Bartholomew's Hospital; Thomas Watts, Manchester and Durham University; Richard Henslowe Wellington, St. Bartholomew's Hospital; Lionel Frederick West, Yorkshire College and General Infirmary, Leeds; Herbert Lorraine Earle Wilkes, Guy's Hospital; and Harry Bowen Williams, St. Thomas's Hospital.

### ATHLETICS.

### Cricket, May 9th.

St. Mary's Hospital v. Carshalton.—This match was played at Carshalton and resulted in a victory for the hospital by 41 runs. The scores for the hospital were H. R. Power, 5; B. Pares, 5; D. d'Esterre, 11; A. E. F. Reer, 9; W. Overend, 22; J. C. S. Matthew, 14 not out; W. P. Craven, 0; L. Oliver, 0; J. B, Yelp, 0; P. Mould, 1; A. Y. Ryall, 5; and extras 8; total, 80. Carshalton made 39 and of these H. Wingett made 10.

*St. Bartholomew's Hospital v. Kensington Park.*—This match was played at St. Quintin Park, Notting Hill. The Hospital went in first and made 130 for eight wickets, H. E. Hoffmeister making 77 of these. Kensington Park then went in and scored 65 for three wickets, G. T. Campbell making 26. The game thus resulted in a draw.

*London Hospital v. Uxbridge.*—This match was played on Saturday at Uxbridge, the Hospital winning by 10 runs. Birt made 61 and B. Jones 21 for the Hospital. For Uxbridge, J. C. Hilbert made 41. Scores: Hospital, 144; Uxbridge, 134.

